# Pharmacogenomics applied to recombinant human growth hormone responses in children with short stature

**DOI:** 10.1007/s11154-021-09637-1

**Published:** 2021-03-12

**Authors:** Adam Stevens, Reena Perchard, Terence Garner, Peter Clayton, Philip Murray

**Affiliations:** Division of Developmental Biology and Medicine, School of Medical Sciences, The Faculty of Biology, Medicine, and Health, University of Manchester, Manchester, UK

**Keywords:** Pharmacogenomics, Transcriptomics, Growth hormone, Interactome

## Abstract

We present current knowledge concerning the pharmacogenomics of growth hormone therapy in children with short stature. We consider the evidence now emerging for the polygenic nature of response to recombinant human growth hormone (r-hGH). These data are related predominantly to the use of transcriptomic data for prediction. The impact of the complex interactions of developmental phenotype over childhood on response to r-hGH are discussed. Finally, the issues that need to be addressed in order to develop a clinical test are described.

## Short stature and r-hGH therapy

Short stature is defined as a height greater than two standard deviations (SD) below the mean for age which is equivalent to less than the 2^nd^ percentile on a standard growth chart [1]. Around 15% of children assessed for short stature will have an identifiable underlying cause (for example growth hormone deficiency, Turner Syndrome, a skeletal disorder or a systemic disease such as coeliac disease) while for the remaining 85% a diagnosis will be given based on describing their growth pattern e.g. Small for Gestational Age (SGA), Idiopathic Short Stature (iSS) or Familial Short Stature [2]. Endocrine or genetic disorders are more likely with increasing severity of short stature.

Treatment with recombinant human growth hormone (r-hGH) is indicated in a range of short stature pathologies [3, 4] including growth hormone deficiency, Turner syndrome, chronic renal impairment, Prader-Willi syndrome, Noonan syndrome, ISS and the child born SGA. The cost of treatment with r-hGH is high: the global growth hormone market for the treatment of growth hormone deficiency (GHD) is currently ~$2 billion and is expected to reach $3 billion by 2022. In the UK the cost of r-hGH treatment has been calculated to be between £6,000 and £24,000 per centimetre gained in final height [5]. Whilst r-hGH is considered to be safe with few adverse events [6], concerns remain with the use of supra-physiological doses of growth hormone during childhood [7].

Response to r-hGH treatment is highly variable [8] depending on the underlying condition and age at initiation of treament [9–11]. The observed variation in response to r-hGH therapy is of particular concern when considered alongside the high cost, burden of injections for the child and potential long-term consequences of treatment. Considering these points predictive models of response to r-hGH are of importance in guiding treatment decisions. Currently prediction is based on linear regression models and can account for up to 60% of the variance in response over a range of conditions [12–14].

## Pharmacogenomics of response to recombinant human growth hormone

Pharmacogenomics is the study of how genetic variation across the whole genome impacts drug response. Obvious candidates for the impact of genetic variation on response to r-hGH are the genes involved in generating the core function [15–17] within the GH-IGF axis. It is of note that whilst genetic variation has been established in these pathways (Table 1) and linked to response to r-hGH treatment, variants in these genes can only account for either a fraction of the effect [18] or, for pathogenic variants, specific familial pathologies with Mendelian inheritance [15]. *GH1* gene deletions, nonsense and frameshift mutations lead to the development of isolated growth hormone deficiency type 1A. In this condition there is a complete absence of GH protein and after therapy with r-hGH is started anti-GH antibodies develop leading to an extremely poor response to treatment [19]. Both endogenous and exogenous r-hGH act via the generation of IGF-I which signals via the IGF-I receptor (*IGF1R*). Mutations in *IGF1R* result in children being born SGA with microcephaly and post-natal growth impairment. SGA children with *IGF1R* mutations when compared to SGA children without such mutations display a lower 1^st^ year response to r-hGH as measured by Δheight velocity SDS or Δ height SDS with 52% classified as poor responders compared to 17% in the control SGA group [20]. Non-additive interaction of the GH Receptor (*GHR*) exon 3 deletion (d3) variant, an *IGF1* variant and an *IGFBP3* variant has been observed [21, 22].

**Table 1:** Genetic variation in core growth pathways associated with response to r-hGH

Gene	Variant	Impact
*GH1*	Various	Isolated GHD [23] with very poor response to treatment in IGHD type 1A
*GHR*	d3	Increase in growth response in first year of therapy by ~1cm [24, 25]
*IGF1*	IGF(CA repeat)19	Homozygosity is associated with less favourable growth outcomes in patients with severe GHD [21]
*IGFBP3*	-202 A/C IGFBP3	Greater height velocity in first year of r-hGH treatment in GHD [22]
*IGF1R*	Various	Lower 1^st^ year growth reponse

Genetic variation associated with response to r-hGH has also been established in biological pathways immediately proximal to and impacted by GH/IGF1 pathways. Genetic variants in *SOCS2*, a negative regulator of GH receptor signalling, were shown to impact adult height standard deviation scores of patients after r-hGH treatment for Turner syndrome (TS) and GHD (up to 0.7 higher) [26]. An association with the vitamin D receptor gene, *VDR*, has been noted [17, 27] but with some contention as to its impact [18]. Genetic variation in the leptin receptor, *LEPR*, has also been implicated in the modulation of response to r-hGH [28].

Using GHD and TS patients a large candidate gene study of response to r-hGH during the first year of treatment has been conducted that examined genetic variation in 103 genes within i) the GH/IGF1 axis, ii) bone and cell growth and iii) glucose and lipid metabolism pathways, as either core growth pathways or pathways immediately related to growth. Eleven genes in GHD and ten in TS, with two overlapping, were associated with first year growth response [29]. The growth response association of four of these genes (*SOS1* and *INPPL1* in GHD and *ESR1* and *PTPN1* in TS) was weakly supported in a validation study using clinical and auxological covariates in regression models [30]. Evidence for association with response was shown in a further five genes (*IGF2*, *GRB10*, *FOS*, *IGFBP3* and *GHRHR*) in severe GHD (≤ 4 μg/L in stimulation test) using machine learning (random forest) [30]. However, it was deemed that the contribution of these variants in a prediction model of first-year response was not sufficient for routine clinical use [30]. In the same initial data set an analysis of growth response over five years of treatment with r-hGH found a range of possible associated genetic variants but none of them were considered to have sufficient power for prediction [31]. It was postulated that the impact of covariates related to the child’s developmental stage, disease severity and geographical location (season and latitude – see Section 6) contributed to the difficulty in prediction [30, 32].

Functional analysis was conducted on some of the genetic variants associated with first year growth response (*IGFBP3*, *CYP19A1*, *SOS1*, *GRB10*) [33]. The genetic variants in these genes were shown to have an additive impact on first year growth response. Reporter gene analysis using the genetic variants demonstrated an impact on transcriptional activity that provided a rationale for the clinical impact [33]. A gene was selected for further analysis, *GRB10*, a modulator of IGF1/insulin signalling pathways. *GRB10* is a negative regulator of growth and one of the genes previously associated with response in severe GHD [34]. Knockdown of the orthologue, *grb10a*, in Zebrafish (*Danio rerio*) resulted in increased animal length thus indicating a clear functional link to growth response [35].

Overall studies on the genetic variation related to *candidate* genes in the core growth and associated pathways has shown small effect sizes resulting in difficulties in replication against the background of contributions from covariates. However, functional analysis and the investigation of additive effects provides supporting evidence of a mechanistic relationship of genetic variants with response to r-hGH. Together these data implicate a complex genetic response that underpins the high variation observed in patients.

## Response to recombinant human growth hormone is polygenic

Recently, a *genome wide* association study (GWAS) on r-hGH response over the first year of treatment has been published [36]. Inevitably this work uses small numbers (614 individuals from 5 short stature cohorts receiving r-hGH: 297 with ISS, 276 with isolated GHD, and 65 born SGA). No evidence of genome-wide significance in primary analysis was found, although there was supporting evidence for a relationship with *B4GALT4*, involved in glycolipid synthesis, and *TBCE*, involved in the folding of beta-tubulin. After secondary analysis including replication there was further evidence to support the following genes [gene symbol, function of encoded product], *ST3GAL6*, a sialyltransferase; *UBE4B*, a ubiquitination factor; *CPOX*, an enzyme of the heme biosynthetic pathway; *CLEC7A*, a pattern-recognition receptor in the innate immune response; *OLR1*, a low-density lipoprotein receptor; *LAPTM4B*, a lysosome associated gene; *NT5DC1*, a deoxyribonucleotidase and *COL10A1*, a collagen. It is notable that the majority of these genes are not obvious growth-related candidates.

This study also showed that previously identified core growth pathway genetic variants associated with response to r-hGH, in *GHR* d3 and *IGFBP3*, were not replicated in this GWAS study. A polygenetic score based on genetic variants associated with final adult height provided no evidence of a relationship. The authors concluded that final height and growth response are not strongly connected. They also postulated that rare, mendelian variants related to short stature could be missed in the analysis. Overall, this work has shown that any heritable contribution to response to r-hGH is likely to be polygenic.

## The role of transcriptomic data in understanding the impact of complex genetics

**Fig. 1:**
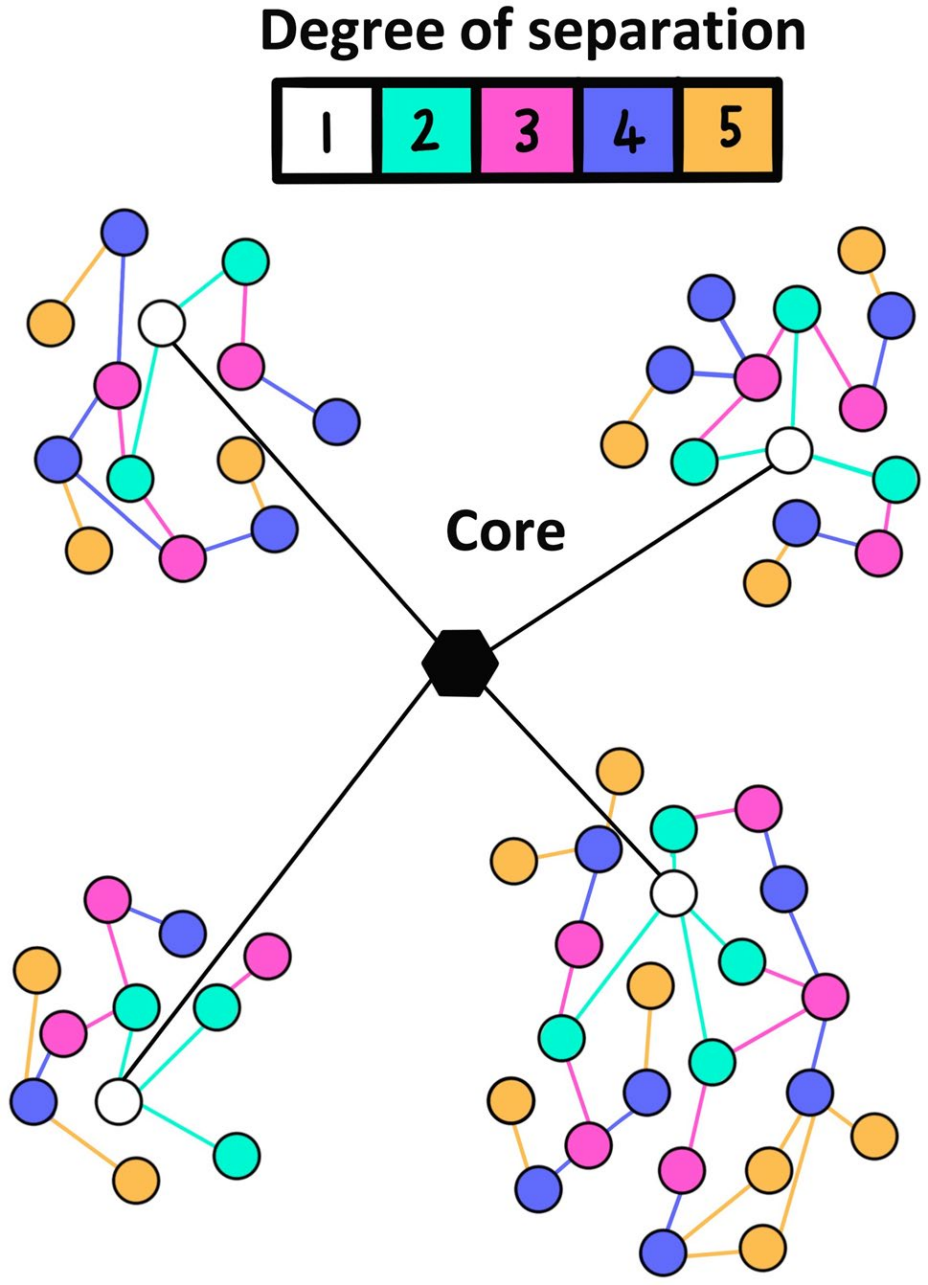
The small world property of network models. Biological networks are characterised by the aggregation of clusters of genes/proteins (nodes) around central core units that deliver key function. The degree of separation of each gene/protein from the core unit is represented by the colour of the node. Local neighbourhoods are represented by clusters of nodes

If response to r-hGH is polygenic then the associated genetic variation is likely to be spread across the genome and include many non-coding variants [37, 38]. It has been proposed that gene regulatory networks have sufficient connectivity that all genes expressed in disease relevant tissue can affect the function of the core pathways that control the condition [39]. This idea has been modelled in human final height GWAS data [40] and it has been shown that the majority of heritability can be explained by genes outside of the core pathways due to the “small world” property of biological networks (Fig. 1). Tissue specificity can be easily related to the traits under investigation using a network model. Databases, such as GTEx [41], can be used to provide tissue-specific gene expression to identify expression quantitative trait loci (eQTL) thus linking gene expression to genetic variants. The authors proposed that overall effect size of any genetic variant would be the weighted average of its effect in each relevant tissue and termed this idea the ‘omnigenic’ model of complex traits [39]. The omnigenic model explains the perceived lack of relationship between the r-hGH response GWAS findings and the expected effect of core growth related pathways.

Transcriptomic data represents the impact of the whole genome in the sampled tissue, and associated network modelling links genes with differential expression to the entire genomic background via the omnigenic hypothesis. In a rare disease, such as GHD, a major problem with genetic investigation is that patient numbers will never be sufficient for a fully powered analysis with GWAS and this is compounded by the possible impact of rare mendelian variants. The transcriptome reflects the combined effect of multiple gene variants on mRNA expression, and the ability to detect an effect will be higher than with GWAS as there will be less adjustment for multiple testing and therefore a greater signal window [42]. Analysis of the transcriptome is likely to achieve robust findings as long as the impact of the source tissue is considered, and careful consideration is placed on the control of phenotypic covariates [43, 44].

## Tissue specificity of the transcriptome: function versus biomarkers

Compared with other tissue samples in adults and children, blood is easily accessible. Blood transcriptomic data can be generated by routine processing. Whilst the functional relevance of the blood transcriptome to a condition under investigation, such as a growth disorder, may be questioned, the presence of blood transcriptome biomarkers may still be relevant. In the case of r-hGH therapy, the blood transcriptome may not indicate a direct functional relationship to what is happening at the growth plates but could be a source of transcriptomic biomarkers that indirectly reflect growth response. We know that peripheral blood monocytes (PBMCs) generate a transcriptomic response to growth hormone [31, 45–47], therefore blood transcriptomic markers of response to r-hGH are possible. Furthermore, an analysis of the blood transcriptome associated with severity of GH deficiency found 271 genes (represented by 347 probe sets) of which 65 were also expressed in the human growth plate (24%). The prediction of GHD from controls was also investigated, of 53 transcriptomic markers with predictive capacity 10 were also expressed in the human growth plate (19% enrichment, $p<1.1\times {10}^{-12}$) [48]. The observation of this significant overlap between the blood and growth plate transcriptomes highlights that even in seemingly very different tissues core elements of key pathways have conserved expression.

In fact, it has been shown at the level of the interactome network (the known protein interactions that occur between the products of expressed genes representing a model of function) that conservation of key pathways is high between different tissues [49]. This observation is further supported by the presence of common gene expression patterns between tissues [50, 51]. These tissue specific elements do not tend to exist in isolation but instead they have specific connections into the conserved core pathways [49]. It has also been observed that genes expressed in a specific tissue tend to localise in the same *neighbourhood* of the interactome (defined as a cluster of proteins with a high density of connections [observe the structure represented in Fig. 1]); it is the integrity of these neighbourhoods that defines the gene signature related to the condition being examined [52].

More recently it has been found that considering tissue level expression in relation to disease phenotype can improve classification of rare disease [43, 53] and that genetic variants that influence gene expression in multiple tissues are more likely to influence multiple complex traits [54]. Taken together these findings show that a tissue seemingly unrelated to a complex phenotype may reflect disease function. Whilst studies claiming functional relevance to a growth phenotype may always be criticised if based solely on the blood transcriptome, the current literature supports the idea that blood transcriptome biomarkers can be used to predict phenotype seemingly unrelated to blood. In the case of response to r-hGH the fact that blood transcriptome has been shown to change in relation to growth hormone exposure suggests a utility that has been unexplored until recently.

## Interactions with developmental phenotype and the environment confound prediction of response to recombinant human growth hormone

The age of a child has been shown to relate to the blood transcriptome in a manner that corresponds to the stages of childhood development (infancy, childhood, puberty) [55]. The limited age-related analysis of the transcriptome that was possible in other tissues demonstrated a tissue independent transcriptomic signature as seen in mouse and rat models corresponding to a co-ordinated whole body genetic program for growth [56–58].

Response to r-hGH has been related to the variation of phenotypic measures using linear models [59]. Key phenotype interactions include the age of the child at start of treatment, parental height, body weight, and birth weight. Analysis based on these data has revealed that younger children tend to respond better to treatment with r-hGH [12–14] implying an interaction of response with the developmental process.

Interactions of genetic variants with the developmental phenotype of children receiving r-hGH treatment have been reported [30, 36]. In the recent growth response GWAS, age and gender were used as covariates in a minimally adjusted model; further growth phenotype related covariates (including birth weight, parental height and gestational age amongst others) were introduced in a maximally adjusted model and shown not to introduce systematic bias but were also shown to yield different results from the analysis [36]. Together these studies imply that an interaction between genetic variants associated with response to r-hGH and multiple growth-related phenotypes occurs. As noted earlier, this observation implies a likely action of the associated genetic variants in multiple tissues [54].

An environmental interaction with growth response related genetic variants has been observed [32]. Summer daylight exposure (SDE), a correlate of latitude, was shown to be of equivalent importance to age in the prediction of growth response. Interaction of genetic variants associated with growth response were observed. A positive relationship of growth response with SDE was seen with carriage of genetic variants in *IGFBP3*, *TGFA* and *TP53*. Conversely a negative relationship was observed in *GRB10* and *CYP19A1*. In this study an association of the blood transcriptome with SDE was defined and linked to growth response.

Unlike genetic variants, that in theory remain constant, variation in the transcriptome is related to both environmental factors and the developmental process itself. This can present both an advantage and disadvantage to the use of transcriptomic data for prediction of response to r-hGH. The advantage is that the impact of complex growth traits is summarised in the levels of gene expression and, therefore, can be viewed as a direct measurement of the combined impact of the genotype, environment, and developmental stage. The disadvantage is that the deconvolution of these phenotypic interactions to identify their relative contributions is difficult as it is confounded by non-linear relationships.

## Transcriptomic data as a tool to investigate response to recombinant human growth hormone

Recently the use of the blood transcriptome in prediction of response to r-hGH has been investigated [31], using an interactome network approach. In GH deficiency (GHD) and Turner Syndrome (TS) patients an identical set of genes was identified whose expression could be used to classify therapeutic response to r-hGH in both conditions with a high accuracy (area under the curve of the receiver operating characteristic [AUC] > 0.9). Importantly, the transcriptomic data were corrected for a range of covariates – microarray batch, age, body mass index (BMI) at baseline for both GHD and TS patients along with gender and peak GH test response in GHD. Tanner stage was also added as a covariate to control for differences in pubertal transition during treatment. The analysis was performed using network models based on the pre-treatment *baseline* transcriptome related to the growth response to r-hGH in each of the five years over which the study was conducted. No predictive gene was present in the core classical growth networks, a few genes were proximal to core pathways (e.g. *GRB2*, predictive of response in the second year of treatment) and most were distant to the core. This result is in alignment with the omnigenic hypothesis although, as discussed previously, care must be taken with functional interpretation from the blood transcriptome in relation to response to r-hGH. None of the genes identified were found as primary findings in the r-hGH response GWAS but the relationship of most of the genes to the core growth pathways is similar to that observed in the GWAS, namely that the genes are distant to the core. The lack of overlap with the GWAS study is likely to be compounded by sample size and group composition, as the GWAS study included a large number of children with ISS and none with TS. Of the 58 genes with expression that had predictive value at any year of r-hGH treatment, seven had also been observed to have a genetic variant related to growth response. Together these data relate the use of the blood transcriptome to the underlying complex genetic landscape.

An achievement of the network modelling used to refine the transcriptomic data associated with prediction of response to r-hGH is that sets of genes were defined that could be used in both GHD and TS to effectively predict both good and poor response. This is notable as the two conditions have a very different genetic background and a higher dose of r-hGH is used to treat short stature in TS. Importantly, this observation implies that a condition independent response to r-hGH is likely.

## The use of epigenomic data in the prediction of response to recombinant human growth hormone and the relevance of newer omic technologies

The DNA methylation based epigenome of PBMCs has been investigated in relation to long-term response r-hGH [60]. The authors concluded that there was little evidence of a coherent change to the epigenome in the context of long-term treatment safety. These data were reanalysed in relation to response to r-hGH and an association of gene region hypermethylation with poor response to r-hGH was identified [31]. The genes with predictive expression in both GHD and TS were shown to be related to DNA methylation using these data [31]. This observation supports the predictive value of gene expression and presents the possibility that epigenomic markers could also be used for prediction.

RNAseq has now largely supplanted microarrays as the choice technique for transcriptomic analysis. RNAseq has the advantage of directly quantifying the sequences present and therefore has a larger and more stable signal window than microarray analysis. Techniques have been derived from RNAseq that present opportunities for further work to define the genomic relationship with response to r-hGH.

Single cell RNAseq can be used to assess the transcriptome in many thousands of cells from the same sample. This approach presents the possibility of mapping differences in response between specific cell types but importantly has also resulted in the generation of two newer approaches that would give specific benefit.

The first is termed RNA Velocity [61, 62]. This approach uses the sequence data that is generated to derive a value for the rate of change of RNA expression. This is based on a ratio between those DNA fragments from the same gene that contain intronic sequences and those that contain just exonic sequence. The ability to work with RNA velocity data would potentially allow the monitoring of response to r-hGH during treatment to assess action. These data would add an extra dimension to using transcriptomic data and may allow the refinement of prediction models.

The second is called expressed variant analysis [63–65]. It has been shown that it is possible to separate cells belonging to different individuals in single-cell RNA-seq runs by analysing the genes that contain a variation from the reference sequence. These differences represent expressed single nucleotide variants (SNVs) and their presence can be used to perform inter-individual normalisation, in addition to enabling further analysis of the data using the variant call results. This approach can be used to assess the impact of expressed genetic variation directly on associated gene expression, similar to an eQTL at a cellular level, and generate a link between the genome and the transcriptome in association with response to r-hGH. Whilst the majority of inter-individual genetic variation may be non-coding, expressed variant analysis identifies the impact of exonic variants including potentially rare mendelian variation. Expressed variant analysis therefore has the potential to identify novel mendelian genetic variants affecting response.

## Towards a clinical test?

Pre-treatment phenotype of short stature patients can be used to predict response to r-hGH using linear modelling approaches [59, 66–68]. In further analysis of the predictive value of transcriptomic data using GHD and TS patients, the baseline clinical parameters alone were shown to achieve good classification of response to r-hGH using random forest (GHD AUC range: 0.86–0.94, TS AUC range: 0.84–0.91) [31]. However, it was also found that adding the blood transcriptome markers to the random forest increased predictive value at each year (GHD AUC range: 0.95–0.97, TS AUC range: 0.92–0.95). This amounted to a small (4–7%) but significant increase in prediction of response to r-hGH. Importantly it was also noted that the transcriptomic markers alone have at least the equivalent predictive value compared to using just the patient phenotype. It is of note that two of the primary variables in the linear modelling are mid-parental height and distance to target height, two variables that can be thought of as surrogates for inter-individual genetic variation.

A further key feature of the predictive modelling using both transcriptomic and phenotypic data was that a significant decrease of error rate was observed in the prediction of growth response at each year when blood transcriptome markers were included. Error rates significantly decreased to an average of 5% in both GHD and TS. This represented a halving of the error rate seen when predicting response to r-hGH using only phenotypic markers [31].

Evidence from linear modelling of phenotype supports the observation that the first year of treatment with r-hGH generates the best response to r-hGH [67, 69, 70], although this observation is likely to be colinear with age. It was noted in the work using the blood transcriptome to predict response to r-hGH that the network model structure changed during the third year of treatment indicating a “gear shift” between earlier and later response to r-hGH over the five years studied [31]. The blood transcriptome work indicated that response at all years of the study could be predicted by the baseline data. These data imply that the early and late responses to r-hGH are modelled at the level of the baseline interactome in PBMCs, and therefore the pre-treatment environment is influencing long-term response. More work is required to unravel the implications of this result.

The GWAS data raise the possibility of a genetic risk score related to response to r-hGH. Whilst this has been postulated by the authors, they also point out that larger sample sizes would be required to generate such an approach [36]. It has been proposed that transcriptomic data can be used to generate risk scores that link eQTLs with GWAS data [71, 72]. This approach has been shown to outperform polygenic risk scores in distinguishing those with Crohn’s disease from healthy individuals [71].

The journey towards developing an accessible genetic/transcriptomic test to support the classification of response to r-hGH for clinical use now requires further evaluation. This would entail a rapid turn-around genomic test combined with an artificial intelligence algorithm. The use of machine learning to support prediction of response can generate worries concerning the “black box” nature of these techniques, although modifications are being developed to improve these problems [73]. However, it is increasingly possible to “deconvolve” the models and drill down to key findings to support the generation of a simple risk score [74].

## Conclusion

The GWAS study combined with the existing pharmacogenomic data now clearly demonstrate that response to r-hGH is largely polygenic [36]. Furthermore, this work demonstrates that growth response to r-hGH is likely to be distinct from the genetic regulation of final adult height.

The case for using transcriptomic data to assess prediction has been developed against the background of a complex genetic response. The network models based on transcriptomic data used to support predictive analysis are like those expected from polygenic conditions. We propose that this link highlights the complex genetic nature of response.

The current utility of linear models to predict response to r-hGH has been set in the context of the genetic findings. The ability of pharmacogenomic and transcriptomic data to provide extra predictive value has been assessed. Further studies will need to be run to confirm the utility of these approaches, but they promise to improve predictive error and also identify the genetic impact implied by mid-parental height and distance to target height measurements.

Future development of the techniques described in this review will provide information on condition specific response. This will be exemplified by the differences in the transcriptome associated with a wider range of growth disorders, including short children born SGA and ISS. These techniques will also define these genomic differences in relation to variants with small effect sizes and novel rare variants with a more mendelian inheritance pattern. As these techniques are refined, differentiation between condition causal and response related genetic variation will be possible.

In conclusion, we have sufficient knowledge of the pharmacogenomic landscape of response to r-hGH to start to move towards development of clinical testing. This is potentially feasible to develop using the blood transcriptome. Further studies will be required to validate these approaches and develop an easy and robust testing strategy.
